# A novel curriculum for the Same-Sex Marriage Act and Patient Right to Autonomy Act (SMPRA) module based on two new laws in Taiwan: a mixed-methods study

**DOI:** 10.1186/s12909-023-04076-9

**Published:** 2023-02-04

**Authors:** Yi-Chih Shiao, Zxy-Yann Jane Lu, Chung-Pei Fu, Jing-Yi Lin, Yaw-Wen Chang, Wan-Ting Chen, Chih-Chia Wang

**Affiliations:** 1 School of Medicine, National Defense Medical Center, No.161, Sec. 6, Minquan E. Rd., Neihu Dist, Taipei City, 11490 Taiwan; 2 Department of Family and Community Medicine, Tri-Service General Hospital, National Defense Medical Center, No.325, Sec. 2, Chenggong Rd., Neihu Dist, Taipei City, 11490 Taiwan; 3 College of Law, National Chengchi University, No.64, Sec.2, ZhiNan Rd., Wenshan District, Taipei City, 11605 Taiwan; 4 Department of Bioscience Technology, Chung Yuan Christian University, No. 200, Zhongbei Rd., Zhongli Dist, Taoyuan City, 320314 Taiwan; 5 Department of Occupational Therapy, College of Medicine, Fu Jen Catholic University, No.510, Zhongzheng Rd., Xinzhuang Dist, New Taipei City, 24205 Taiwan; 6 Department of Psychiatry, Tri-Service General Hospital, National Defense Medical Center, No.325, Sec. 2, Chenggong Rd., Neihu Dist, Taipei City, 11490 Taiwan

**Keywords:** Gender, Medicine, Law, Sexual rights, Medical ethics, Legal ethics

## Abstract

**Background:**

The establishment of laws has had a tremendous impact on holistic medical care. The Patient Right to Autonomy (PRA) Act and the Same-Sex Marriage Act have been passed in Taiwan, and both have sparked intense societal debate. The *S*ame-Sex *M*arriage Act and *PRA* Act (SMPRA) teaching module was created for the Gender, Medicine, and Law (GML) course of the medical curriculum. This video trigger-assisted problem-based learning (VTA–PBL) software has integrated content on the aforementioned legislative proclamations. It upends conventional beliefs and fosters reflective practices on sexual rights and the right to representation among medical students. This study examined how the SMPRA module affected the knowledge and attitudes of medical students taking up the GML course.

**Methods:**

A simple pre-/post-test design evaluated the outcomes of the PBL module to examine the changes in knowledge and attitudes of medical students toward same-sex marriage rights. In 2019 and 2020, 126 and 49 5th-year medical students took up the GML course, respectively. The GML components included a video scenario representing advanced decision-making and a healthcare agency with a same-sex couple, a PBL discussion, and student feedback presentations. The mechanisms of feedback collection and measuring student knowledge and attitudes toward sexual rights differed between one cohort in 2019 and the other in 2020. Pre- and post-lecture tests were used in the first school year, whereas a post-lecture open-ended questionnaire survey was used in the second school year.

**Results:**

In total, 90 and 39 eligible questionnaires were received in the first and second school years, respectively, which corresponded to response rates of 71% and 80%. Students showed a better understanding of and positive enhancement of proficiency in legal and ethical content and relevant clinical practice. Qualitative analysis revealed that students viewed healthcare providers as checkpoints for conflicts of interest; medical ethics as the cornerstone of clinical practice; cultural background as a significant influence on decision-making; and empathetic communication as the cornerstone of relationships between patients, family members, and doctors.

**Conclusion:**

The GML course of the SMPRA module fosters reflective practices on ethical and legal sexual rights issues.

**Supplementary Information:**

The online version contains supplementary material available at 10.1186/s12909-023-04076-9.

## Background

A significant step forward was made in the advancement of human rights in Taiwan in 2019 with the adoption of the Patient Right to Autonomy (PRA) Act and the Enforcement Act of Judicial Yuan Interpretation No. 748 (Same-Sex Marriage [SSM] Act) by the Constitutional Court of Taiwan. These new laws have sparked a great deal of social debate; they challenge traditional values and highlight the need for reflective practices regarding sexual rights. These pioneering judicial works in Taiwan are based on the Sustainable Development Goals, including gender equality, peace, and justice [1].

By bringing medical care closer to the primary framework of patients’ rights, minimizing discrimination, and protecting mental health, the protection of PRA and SSM rights (SSMR) enables patients to experience substantial benefits [2]. Favorable changes in medical services usage and spending have been observed in gender minority groups following the passing of the SSM Laws demonstrating the positive benefits of the SSM legislation [3]. On the contrary, negative influences with immediate mental burden and avoidance of appropriately in-time access to medical services among gender minorities could be learned from the historical context in Nigeria with the Same-Sex Marriage Prohibition Act passed in 2014 [4].

Holistic medical care has been significantly impacted by the enactment of laws. However, the teaching framework for the current medical education system does not yet include the PRA and SSM Acts. Future clinicians' unfamiliarity with the new updated information in the advanced decision (AD) and healthcare agent (HCA) systems may not only jeopardize the rights and interests of patients but also lead to moral dilemmas. Therefore, the issues related to gender and medical law need integration into the curriculum to facilitate medical students’ reflection on gender norms and gender identity. To promote the development of empathy and ethical judgment among medical students, one of the writers (WCC) created the Gender, Medicine, and Law (GML) course for undergraduate 5^th^-year medical students in their family medicine rotation.

Because of the lack of existing teaching resources for medical students in Taiwan, the creator of the GML course invested in the creation of new materials following the National Government's promulgation of the SSM and PRA Acts and facilitated student discussion utilizing video trigger-assisted PBL (VTA–PBL). The objectives of this study were to investigate the effects on students’ performance and proficiency with SSMR and PRA in 2019 and to evaluate their perspectives toward the GML course in 2020.

In Taiwan, it is innovative to include SSMR and PRA in the teaching curriculum for medical students. Based on a number of the benefits of video triggers, the GML utilizes a problem-based learning (PBL) technique, including avoiding patient depersonalization, observing doctor–patient interactions, and increasing motivation to solve problems [5], to ensure it helps students learn and be able to thoroughly debate the problems at hand. The VTA–PBL curriculum design focuses on describing and highlighting communication, dialogue, and access to the patient’s situation [6] to enhance the empathy of medical students in comparison with a text-oriented curriculum design.

## Methods

### The *S*ame-Sex *M*arriage Act and *PRA* Act (SMPRA) module

PBL has been widely employed in medical education since 1986 [7]. With the development of hardware technology, PBL has incorporated video trigger assistance, enabling students to examine clinical settings up close [5]. VTA–PBL has been found to increase student engagement [8] and to allow students to understand the complexity of clinical dilemmas [5]. Based on the benefits of VTA–PBL outlined above, the curriculum in this article was designed to follow it. In Taiwan, the SSMR and PRA requirements were implemented early, but there are no ready-made video teaching materials yet that are pertinent. Our research team produced the teaching content for the GML course, referred to as the combined SMPRA module, in a two-stage manner. In the first stage, a video scenario was created by our research team after Judicial Yuan Interpretation No. 748 was released (May 24, 2017). A lecturer asked undergraduate medical students to assist in writing a script (see Additional file 1) and filming scenario highlighting potential medical problem after the emergence of the SSM and PRA Acts and to participate in filming the scenario. In the second stage, the VTA–PBL curriculum design was executed.

### Study design and settings

**Fig. 1:**
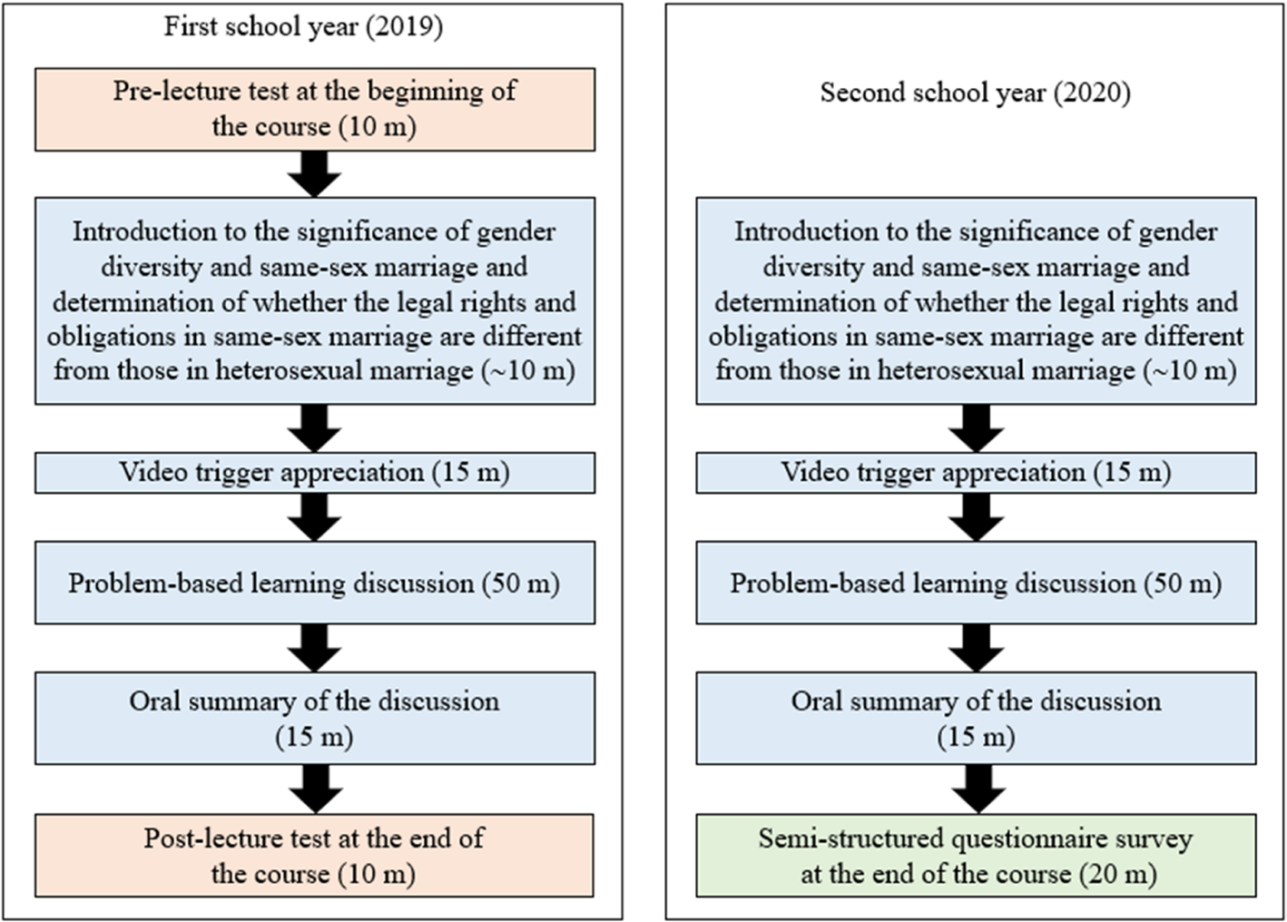
Gender, Medicine, and Law course flow. The lecture on Gender, Medicine, and Law (GML) comprised 10 min of introduction, 15 min of video trigger appreciation, 50 min of the PBL discussion, and 15 min of oral presentation. Pre-test and post-test were conducted at the beginning of 5 min and the ending of 5 min of the class in the first school year. Different mechanisms of feedback collection were used in the first and second school years to measure student performance, knowledge, attitudes, and perspectives. The prior one was pre-/post-lecture tests, and the latter one was a post-lecture semi-structured questionnaire

This research study was authorized by the Tri-Service General Hospital Review Board (approval no. B-108–04). Medical students’ knowledge and attitudes toward SSMR changed because of the PBL program, which was evaluated using a straightforward pre-/post-test approach. The GML course contains the following four components: [1] introduction to the legal rights and obligations stipulated in the SSM and PRA Acts (~ 10 min); [2] video trigger appreciation (~ 15 min); [3] PBL discussion (~ 50 min); and [4] oral summary of the discussion presented by the students at the end of the course (~ 15 min) (Fig. 1). The outcomes of the GML course utilizing the SMPRA module were measured using two assessment instruments that maintained anonymity and were taken willingly by the participants. The first tool, a pre-/post-test self-assessment questionnaire (SAQ) was administered during the first and last 10 min of class in the 2019 academic year to assess their proficiency and understanding in relation to SSMR and PRA. In the final 20 min of class in the 2020 academic year, a semi-structured questionnaire about the student's classroom experiences on the course and other ideas pertinent to the GML field was administered.

### Participants

Our study participants were fifth-year medical students in their family medicine rotation, a required course for 5th-year medical students in Taiwan, which has seven students in each stratum and awards two credits. They must take the GML course as a requirement for their rotation. From August 2019 to May 2020, the first year of this project, 126 students were included; in the second year, 49 students were involved from January 2021 to April 2021. All enrolled students provided their responses and gave their informed consent to participate in this study.

### Instruments

The quantitative SAQ comprised the following two parts: The first part (SAQ-1–Proficiency; Additional file 2) has six questions each rated on a five-point Likert-type scale (1 = poor understanding, 2 = fair understanding, 3 = average understanding, 4 = very good understanding, or 5 = excellent understanding). The second part (SAQ-2–Performance; Additional file 3) is composed of six true/false questions that measure performance. The two open-ended items in the semi-structured questionnaire about classroom experience (Additional file 4) were used to qualitatively assess the participants’ perspectives on SSMR and PRA. Written comments were collected. The GML course developer provided clear instructions on writing guidance for the students, set a 3-day deadline to receive feedback, ensured their anonymity, and assured them that their feedback would not influence their grades. Two teaching assistants helped collect information, conduct de-identification, and screen samples for eligibility. The idea of double screening, normally used to create systematic-review articles with less bias and lower amounts of missing relevant data inspired the technique of using two assistants to screen eligible samples rather than just one [9]. Feedback lacking content, content beyond GML scope, and feedback that failed to meet the 3-day deadline were considered invalid. The contents of the remaining eligible questionnaires were examined.

### Analysis

This research study applied the mixed-methods evaluation strategy to analyze the data [10]. SPSS was employed to evaluate quantitative data using a two-tailed paired *t*-test (SPSS Inc., Chicago, Illinois, United States). Reliability of the questionnaire was analyzed for internal consistency using Cronbach's α coefficient. Value of Cronbach's α coefficient greater than or equal to 0.70 was regarded to be satisfactory [11]. Qualitative data were analyzed using thematic analysis [12], and an inductive method [13] was used in the following sequence to examine qualitative data. First, the five longest feedback points we chose and carefully examined in our sampling bank to find pertinent words and phrases regarding the goals of this study. Subsequently, these were rudimentarily coded by two of our researchers with interdisciplinary professional backgrounds in medicine and medical law. Second, our researchers achieved preliminary consensus on the coding classification through comparison, discussions, and integration of all reviewers’ codes. A third researcher partook in the review once disagreement occurred. The final agreement with a category code without any new code emerging was reached by the researchers to analyze the remaining data. To ensure the correctness of the original language, all data originally written in Chinese were translated into English and proofread by native English speakers.

## Results

### Quantitative analysis

In the school year of 2019, 126 questionnaire returns yielded 90 valid responses for a response rate of 71%. Internal consistency was satisfactory, as evidenced by Cronbach's α of the SAQ-1–Proficiency (α = 0.88) and the SAQ-2–Performance (α = 0.70). In terms of the students’ proficiency (Table 1), self-assessed scores on the following six items in the post-lecture SAQ were higher than those in the pre-lecture SAQ were, as follows: Q1, Meaningfulness of gender diversity and its relevance to healthcare (3.79 ± 0.57 vs. 3.08 ± 0.62; *t* =  − 10.00; *p* < 0.001); Q2, Legalization of same-sex couples to proceed into a permanent and exclusive status (3.80 ± 0.57 vs. 3.11 ± 0.63; *t* =  − 9.84; *p* < 0.001); Q3, Ethical and legal aspects of signing formal medical documents (e.g., surgical consent) by the same-sex partner (3.88 ± 0.49 vs. 2.94 ± 0.69; *t* =  − 11.86; *p* < 0.001); Q4, The process for signing formal medical documents (e.g., surgical consent) by the same-sex partner (3.74 ± 0.61 vs. 2.59 ± 0.67; *t* =  − 13.60; *p* < 0.001); Q5, The morality and legality of a same-sex partner's signature about the rejection of life-supporting care (e.g., cardiopulmonary resuscitation [CPR]) (3.82 ± 0.57 vs. 2.76 ± 0.69; *t* =  − 13.84; *p* < 0.001); and Q6, The process of AD fulfilled by the same-sex partner against life-sustaining medical care (e.g., cardiopulmonary resuscitation) (3.78 ± 0.63 vs. 2.67 ± 0.70; *t* = -14.22; *p* < 0.001). Regarding the component of knowledge performance about gender diversity, the post-lecture scores were significantly higher than the pre-lecture ones (75.02 ± 17.40 vs. 54.87 ± 14.08; *t* =  − 10.56; *p* < 0.001).

**Table 1:** Results of part 1 of the self-assessment questionnaire (Likert scale): SAQ-1–Proficiency

Item	Pre-test		Post-test		*t*	*p*
	Mean	Standard deviation	Mean	Standard deviation		
Q1: Meaningfulness of gender diversity and its relevance to healthcare	3.08	0.62	3.79	0.57	-10.00	0.000
Q2: Legalization of same-sex couples to enter into a permanent and exclusive status	3.11	0.63	3.80	0.57	-9.84	0.000
Q3: Ethical and legal aspects of signing formal medical documents (e.g., surgical consent) by the same-sex partner	2.94	0.69	3.88	0.49	-11.86	0.000
Q4: The process for signing formal medical documents (e.g., surgical consent) by the same-sex partner	2.59	0.67	3.74	0.61	-13.60	0.000
Q5: The ethics and legitimacy of the same-sex partner’s signature regarding the refusal of life-sustaining treatment (e.g., cardiopulmonary resuscitation)	2.76	0.69	3.82	0.57	-13.84	0.000
Q6: The process of advance decision fulfilled by the same-sex partner against life-sustaining medical care (e.g., cardiopulmonary resuscitation)	2.67	0.70	3.78	0.63	-14.22	0.000

### Qualitative analysis

A total of 49 open-ended responses were obtained, and 39 eligible responses were retrieved with a response rate of 80%. In our assessment, all students responded on the premise that they agreed with the legitimacy of SSMR. More than three-quarters of the students believed that considering the local culture and legal norms is the key in determining the appropriate treatment for individual needs on the premise of full application to the medical profession and professional ethics of physicians. Specific courses in the field of medical law training are indispensable in achieving the proficiency mentioned before. Because the VTA–PBL curriculum design could replicate the medico-legal contexts that they would encounter in the future, the students were happy with it. They concurred that a full understanding of medical law is integral to being a clinician.

The following themes were identified on the basis of the students’ feedback:

1. An excellent model to stimulate students' motivation for inner reflection.
  The VTA–PBL method, which enables students to reflect on their values and viewpoints, was mentioned by the students as being very contemplative and inspirational.
  Few medical ethics-centered PBL courses in the current medical school system exist. Through students’ various conclusions and debates, diverse viewpoints and ideas rooted in observations of simulated scenarios can be inspired. (Student 002)
2. Examine patients in a holistic manner.
  For medical students with less than 1 year of clinical experience, this course is not to accelerate their pursuit of cutting-edge medical knowledge but to help them focus on patients with greater concern about humanistic issues and in depth.
  Taking this course as a starting point, in the future, I should pay attention to the patient and the disease equally, rather than magnify the disease and ignore the patients themselves. (Student 019)
3. Holistically provide healthcare services.
  The provision of professional healthcare services is not only in compliance with medical routines or limited to the indications specified by the guidelines but also in accordance with the needs of patients/families and reach a physician–patient consensus by medical personnel through physician–patient communication (PPC).
  Medical professionals must understand the concerns of patients and their families and encourage dialogue and contact with them to ensure that healthcare services delivered are more in line with patients' and families' preferences. (Student 018)
4. Social acceptance in actual operation would make it harder to apply laws in practice.
  When the development of legal norms considerably outpaces social acceptance, it is easy to encounter a cognitive gap. Conflicts would be foreshadowed if such a gap were not properly closed by shortening conceptual distance.
  Although same-sex couples have become their partners’ HCAs and can make legitimate decisions on their behalf, in Taiwan, where traditional cultural values are strongly ingrained, parents find it difficult to accept such a loss of parental authority. (Student 007)
5. Medical personnel with relevant legal expertise can enhance the sensitivity of spotting legal issues related to patients' rights and become a crucial checkpoint for medical decision-making.
  In addition to providing clinical professional services, medical personnel could clarify the rights and interests of patients as a gatekeeper because the HCA and the insurance beneficiary may be the same person.
  To provide relief to the patient from pain brought on by resuscitation, the HCA decided to employ the power of delegation of authority in clinical situations. However, the medical staff eventually learned that the HCA was the beneficiary of the insurance. The legitimacy issues of the HCA were worth further discussion. (Student 022).
  Apart from the declarant’s heirs, the following people are not eligible to be an HCA under the inclusion and exclusion criteria of the PRA Act's Article 10: the declarant’s legatees, legatees of the declarant’s remains or organs, and other persons who shall benefit from the death of the declarant. Students investigate many facets of how laws are denoted in the class. The discussions on the GML course also guarantee that there will be fewer mistakes in interpretation in the future, hence lowering the prevalence of conflicts of interest.
  Sensitivity to social or cultural contexts would be fostered and adequately promoted if the fundamental ideas and specifics of the legal application of the SSMR and PRA are taught to medical professionals through specialized courses such as the GML as we observed today. Carefully scrutinizing each link by the medical staff (gatekeepers) (e.g., doctors, nurses, or therapists) can reduce the risk of conflict. (Student 005)
6. Acquaintance with the law is necessary.
  All staff members participating in the whole medical community, from undergraduate students to frontline medical personnel, need to be knowledgeable about legal concerns related to medicine and its derivatives. Education is a key to fixing the missing parts in the current Taiwan medical education system.
  Appropriately college education and continuum education for teaching medical law can allow beginners and seasoned workers to recognize potential issues of law in medical environments. The main goal is to ensure that medical practice in accordance with medical routines, applicable laws, and regulations is received by patients, and the secondary effect is to prevent medical disputes brought on by errors made at any step of the medical treatment process. (Student 030)

## Discussion

The fundamental goal of the GML course is to provide undergraduate students with the tools they need to critically evaluate their understanding of and attitudes toward the three subject areas (gender, medicine, and law) it encompasses. Students understand that social and cultural variables, especially sexual rights, are to be integrated into medical decision-making in short order. Collaboration between medical and legal professionals helps reduce the likelihood of conflicts of interest that may arise from identity overlap. The SMPRA module encourages various viewpoints on sexual rights, mortality, and dying while pulling students' attention away from diseases. It considerably contributes to the recognition of effective PPC as humanistic medical care.

In this research study, the students began the deliberation that stemmed from the standpoint of agreeing with SSMR. The students' opinions were consistent with earlier research's finding that medical students could lessen discrimination against sexual orientation after undergoing medical school training [14]. Based on the aforementioned premise, we are convinced that these prospective physicians poised to be on the frontlines of providing medical care services will possess gender-friendly capabilities of communication and mutual understanding.

The AD, proposed in 1967, is the practice of protecting the rights and interests of patients [15]. However, patients’ desires might be compromised by misunderstandings of the notion of AD and the application of knowledge about relevant laws and standards. Tesfa et al. [16] noted that due to major communication issues with healthcare professionals in decision-making, some surrogates do not fully comprehend the roles, responsibilities, and legal authority of HCAs. The feedback from our participants reveals that the trainees’ concepts toward the legal implications and essence of HCAs have transformed from being unclear to being better understood. The study findings depict the SMPRA module as a warm-up routine that adequately gets aspiring medical professionals ready for their admission into treacherous clinical practice settings.

The legal perspectives of the capacity to make judgments and give consent to medical management for healthcare providers are complex, but they could be resolved by systemic education programs [17]. Students are allowed to consider their prior knowledge and attitudes toward sexual rights using VTA–PBL to create simulated situations [18]. Reflective learning is reportedly facilitated in the process of exploring various possibilities and problem-solving skills [19], and it can help students not only develop professional images associated with the simultaneous preservation of core ethical values [20] but also accept the obstacles inherent to their profession [21].

Interpersonal and communication skills are one of the six essential competencies the Accreditation Council for Graduate Medical Education has recognized for doctors. These abilities are the cornerstone of creating a strong doctor–patient relationship [22]. Healthy relationships lacking communication barriers between doctors and patients allow patients to actively participate in decision-making [23], enhance levels of family satisfaction [24], and ameliorate family bereavement outcomes [24]. However, overestimation of the capabilities of PPC among clinicians could be a serious issue [25]. The SMPRA module could be the stimulating point at which students *know*, which is the basis of Miller’s pyramid [26], hence equipping them to better understand patients’ needs, provide genuine empathy in positive perspectives, lower risks of disputes [27], and ultimately *do* what a clinician should do seamlessly [26].

Before 2013, Taiwan had a 7-year medical school system. Since 2013, it has been shortened to a 6-year system due to the implementation of Taiwan’s Taskforce of Medical School Curriculum Reform [28], which had an adverse effect on the teaching effectiveness of human rights and medical humanities [29]. The lack of sufficient teaching time is addressable by including the GML course in family medicine rotation [30]. The next issue that Taiwan faces is how to integrate GML into the medical school program as the consolidation of different professional knowledge areas has posed a great challenge for educators since the 1930s [31]. Our proposed curriculum design could be one of the countermeasures for this problem based on effective brainstorming between students post-VTA–PBL simulation.

Only the implementation has ever been different in single-faceted courses. For instance, in law classes, a transition from legal to medical education has occurred, allowing law students to enter the medical field and discuss patient rights from a legal perspective [32]. Contrarily, medical students are continually exposed to traditional moral conundrums, such as discussions about the appropriateness of abortion or the right to euthanasia [33]. The teaching content is limited to moral elaboration alone [34]. In the gender issues teaching field, there is a huge disparity between teaching styles and ways in which gender issues are covered [30, 35]. Our course design integrates the shortcomings of the past, produces a GML training course, and introduces it into the medical education system as the pertinent laws (the SMR and PRA Acts) were passed to help education conform to the progressive trend of the times.

In recent years, there has been a positive trend in the global attitude toward the lesbian/gay/bisexual/transgender (LGBT) community [36]; Taiwanese society has followed a similar trajectory of change after the lifting of martial law [37]. The acceptance of homosexuality, however, still differs greatly between Asian and Western societies due to different cultural backgrounds combined with extremely diverse values and religious factors [38]. The GML course can be deemed a respite for medical students during their clerkship, allowing them to reflect on the essence of medicine. Physicians can pull the structural level of approaching patients from organic to biopsychosocial and adopt a more comprehensive blueprint for medical care the moment they shift the attention back from the ailment to the patient and family.

This research study has some limitations. It is impossible to completely rule out the practice effect, which could have caused the improvement in the rate of correct answers after the lecture. Additionally, long-term parameters, such as changes in students' behaviors and attitudes toward this issue or relevant challenges, can be difficult to extrapolate because students are dispersed in different groups with diverse schedules of clinical rotations after they complete family medicine rotation. To gather information regarding long-term changes in knowledge, attitudes, behaviors, competency, and skill transformation for analysis of future studies, a cohort-tracking system with measurements via, for example, semi-constructed interviewing and/or post-training tests should be established.

## Conclusion

This study is an appropriate reaction to recent legal concerns closely tied to medical care in Taiwan. The study used VTA–PBL to conduct instruction and transformed legal connotations into course materials. Our research team discovered that students have favorable opinions about this teaching method, which stimulates self-reflection. The SMPRA module promotes the recognition of social and cultural factors as integral parts of humanistic medical care. The medical and law professions are complementary to one another, and their interdisciplinary collaboration can lower risks in clinical practice. Students eventually return to the core purpose of doctors, to save lives and focus on patients as holistic individuals as they work through the process of learning GML.

## Supplementary Information

**Additional file 1. **Video trigger script.

**Additional file 2. **Part 1 of the self-assessment questionnaire (Likert scale): SAQ-1–Proficiency.

**Additional file 3. **Part 2 of the self-assessment questionnaire (true/false): SAQ-2–Performance.

**Additional file 4. **Semi-structured questionnaire about the classroom experience.

## Data Availability

The dataset(s) supporting the conclusions of this article is (are) included within the article (and its additional file(s)).

## Abbreviations

AD: Advance decision
GML: Gender, Medicine, and Law (course)
HCA: Healthcare agent
PBL: Problem-based learning
PPC: Physician–patient communication
PRA: Patient right to autonomy
SAQ: Self-assessment questionnaire
SAQ-1–Proficiency: Part 1 of the self-assessment questionnaire
SAQ-2–Performance: Part 2 of the self-assessment questionnaire
SMPRA: *S*Ame-Sex *M*arriage Act and *P*atient *R*ight to *A*utonomy Act (module)
SSM: Same-sex marriage
SSMR: Same-sex marriage rights
VTA: Video trigger-assisted
