# Correction to: A novel curriculum for the same-sex marriage act and patient right to autonomy act (SMPRA) module based on two new laws in Taiwan: a mixed-methods study

**DOI:** 10.1186/s12909-023-04360-8

**Published:** 2023-05-24

**Authors:** Yi-Chih Shiao, Zxy-Yann Jane Lu, Chung-Pei Fu, Jing-Yi Lin, Yaw-Wen Chang, Wan-Ting Chen, Chih-Chia Wang

**Affiliations:** 1 School of Medicine, National Defense Medical Center, No.161, Sec. 6, Minquan E. Rd., Neihu Dist, Taipei City, 11490 Taiwan; 2 Department of Family and Community Medicine, Tri Service General Hospital, National Defense Medical Center, No.325, Sec. 2, Chenggong Rd., Neihu Dist, Taipei City, 11490 Taiwan; 3 College of Law, National Chengchi University, No.64, Sec.2, ZhiNan Rd., Wenshan District, Taipei City, 11605 Taiwan; 4 Department of Bioscience Technology, Chung Yuan Christian University, No. 200, Zhongbei Rd., Zhongli Dist, Taoyuan City, 320314 Taiwan; 5 Department of Occupational Therapy, College of Medicine, Fu Jen Catholic University, No.510, Zhongzheng Rd., Xinzhuang Dist, New Taipei City, 24205 Taiwan; 6 Department of Psychiatry, Tri Service General Hospital, National Defense Medical Center, No.325, Sec. 2, Chenggong Rd., Neihu Dist, Taipei City, 11490 Taiwan

**Correction: BMC Medical Education (2023) 23:91**

10.1186/s12909-023-04076-9.

Following publication of the original article [1], the authors informed us that there is a typographical error about the description of the number of participants in the “Participants” section.

The original article has been corrected.
