# Supplementary material for: A novel curriculum for the Same-Sex Marriage Act and Patient Right to Autonomy Act (SMPRA) module based on two new laws in Taiwan: a mixed-methods study
Source: BMC Med Educ. 2023 Feb 4;23:91. doi: 10.1186/s12909-023-04076-9 (PMC9899378; doi:10.1186/s12909-023-04076-9)
Supplement: Supplementary file 1 — Additional file 1. Video trigger script. [file 12909_2023_4076_MOESM1_ESM.docx]

**Additional file 1:** Video trigger script.

| The story of the film is centered on a same-sex couple, Ann and Chiao. The same-sex couple had been dating for five years. During the dating period, Chiao’s parents accepted their relationship with an open mind, but Ann did not inform his traditional parents for fear of being blamed. One day, after Ann returned home, he summoned up the courage to tell his mother. The heartbreaking response was the same as he expected. In addition, she argued with Ann and slapped Ann’s face. Heartbroken Ann stormed out of the house and, a traffic accident struck him. After being sent to the hospital by ambulance, Ann’s condition looked very serious, and no more life-sustaining treatment (LST) was recommended, as assessed by doctors. Due to encountering a circumstance of decision-making, the hospital simultaneously contacted Ann’s mother and Chiao as well as the healthcare agent who signed on the document of Ann’s Advance Decision (AD). Nevertheless, only Chiao was successfully reached. Chiao signed the consent form for refusal of LST based on intentions annotated in the AD. Ann’s mother became furious afterward about this decision without any type of notification and decided to file an appeal. Besides, Ann’s mother incidentally found out that Chiao was the insurance beneficiary of Ann’s policy while preparing for a lawsuit. This conflict of interest strengthened Ann’s mom’s belief that insurance benefits influenced the decision made by Chiao. |
| --- |
