# Supplementary material for: A novel curriculum for the Same-Sex Marriage Act and Patient Right to Autonomy Act (SMPRA) module based on two new laws in Taiwan: a mixed-methods study
Source: BMC Med Educ. 2023 Feb 4;23:91. doi: 10.1186/s12909-023-04076-9 (PMC9899378; doi:10.1186/s12909-023-04076-9)
Supplement: Supplementary file 2 — Additional file 2. Part 1 of the self-assessment questionnaire (Likert scale): SAQ-1–Proficiency. [file 12909_2023_4076_MOESM2_ESM.docx]

**Additional file 2:** Part 1 of the self-assessment questionnaire (Likert scale): SAQ-1–Proficiency.

|  | Poor understanding  (1) | Fair understanding  (2) | Average understanding  (3) | Very good understanding  (4) | Excellent understanding  (5) |
| --- | --- | --- | --- | --- | --- |
| Q1: Meaningfulness of gender diversity and its relevance to healthcare |  |  |  |  |  |
| Q2: Legalization of same-sex couples to enter into a permanent and exclusive status |  |  |  |  |  |
| Q3: Ethical and legal aspects of signing formal medical documents (e.g., surgical consent) by the same-sex partner |  |  |  |  |  |
| Q4: The process for signing formal medical documents (e.g., surgical consent) by the same-sex partner |  |  |  |  |  |
| Q5: The ethics and legitimacy of the same-sex partner’s signature regarding the refusal of life-sustaining treatment (e.g., cardiopulmonary resuscitation) |  |  |  |  |  |
| Q6: The process of the advance decision fulfilled by the same-sex partner against life-sustaining medical care (e.g., cardiopulmonary resuscitation) |  |  |  |  |  |
