# Supplementary material for: A novel curriculum for the Same-Sex Marriage Act and Patient Right to Autonomy Act (SMPRA) module based on two new laws in Taiwan: a mixed-methods study
Source: BMC Med Educ. 2023 Feb 4;23:91. doi: 10.1186/s12909-023-04076-9 (PMC9899378; doi:10.1186/s12909-023-04076-9)
Supplement: Supplementary file 3 — Additional file 3. Part 2 of the self-assessment questionnaire (true/false): SAQ-2–Performance. [file 12909_2023_4076_MOESM3_ESM.docx]

**Additional file 3:** Part 2 of the self-assessment questionnaire (true/false): SAQ-2–Performance.

| 1. The term “LGBT” refers to the following components, including lesbians, gays, bisexuals, and transgender people. All of the aforementioned elements belong to the issue of sexual orientation rather than simply biological gender. |
| --- |
| 2. According to Interpretation No. 748 of the Judicial Yuan, same-sex couples, i.e., two persons of the same sex, may form a permanent union of intimate and exclusive nature to live a common life and shall be regarded as spouses by applying the relevant provisions of the Civil Law mutatis mutandis. |
| 3. If a same-sex person becomes a spouse of a couple, the use of assisted reproductive technology could be applied to him (or her) by a medical institution following the provisions of the Artificial Reproduction Act. |
| 4. Two same-sex individuals who are in a relationship, living together, but not registered as a same-sex couple, could mutually sign the consent form for surgery or anesthesia in person through the identity of a partner as one of them is unconscious. |
| 5. Two same-sex individuals who are in a relationship, living together, but not registered as a same-sex couple, could mutually sign the consent form for refusal of cardiopulmonary resuscitation or life-sustaining treatment in person through the identity of a partner as one of them is in the terminal stage of a disease diagnosed by doctors. |
| 6. Two same-sex individuals who are in a relationship, living together, but not registered as a same-sex couple, could mutually designate each other to be their healthcare agent who received written authorization from a declarant to express their wishes on their behalf when they are unconscious or unable to clearly express their wishes. |
