# Supplementary material for: A novel curriculum for the Same-Sex Marriage Act and Patient Right to Autonomy Act (SMPRA) module based on two new laws in Taiwan: a mixed-methods study
Source: BMC Med Educ. 2023 Feb 4;23:91. doi: 10.1186/s12909-023-04076-9 (PMC9899378; doi:10.1186/s12909-023-04076-9)
Supplement: Supplementary file 4 — Additional file 4. Semi-structured questionnaire about the classroom experience. [file 12909_2023_4076_MOESM4_ESM.docx]

**Additional file 4:** Semi-structured questionnaire about the classroom experience.

| 1. Please write down two topics or issues that you are most interested in in this course and explain why. |
| --- |
| (01) First topic or issue: ___________________________________________________________________  Reasons:  (02) Second topic or issue: _________________________________________________________________  Reasons: |
| 2. Please write down your thoughts and suggestions on this course.  Answer: |
